# Reconstruction Methods and Complications of Esophagogastrostomy and Jejunal Interposition in Proximal Gastrectomy for Gastric Cancer: A Meta-Analysis

**DOI:** 10.1155/2020/8179254

**Published:** 2020-01-16

**Authors:** Nan Du, Pei Wu, Pengliang Wang, Yuwei Du, Kai Li, Zhenning Wang, Huimian Xu, Zhi Zhu

**Affiliations:** Department of Surgical Oncology, Department of General Surgery, First Affiliated Hospital, China Medical University, Shenyang, China

## Abstract

**Background:**

Proximal gastrectomy is used for the treatment of primary gastric cancer by open or laparoscopic surgery in the upper third of the stomach. Esophagogastrostomy (EG) or jejunal interposition (JI) is widely used in various reconstruction methods after proximal gastrectomy. We conducted a meta-analysis of EG and JI for treatment of gastric cancer.

**Materials and Methods:**

A search of PubMed, Embase, MEDLINE, J-STAGE, and Cochrane Library identified retrospective series on EG and JI. Weight mean differences (WMDs), odds ratios (ORs), and 95% confidence intervals (CIs) were used to analyze the operation-related data and postoperative complications. Heterogeneity was evaluated by the *I*^2^ test, and potential publication bias was assessed with Egger regression tests and sensitivity analysis.

**Results:**

Eight studies were selected, and 496 patients were included. EG group benefits were 44.81 min shorter operating time (*P* < 0.001), 56.58 mL less blood loss (*P* = 0.03), and 7.4 days shorter hospital stay time (*P* < 0.001) than the JI group. Between the two groups, there was no significant difference in anastomotic leakage; otherwise, the EG group had a lower risk of anastomotic stenosis (OR = 0.44, 95%CI = 0.20 to 0.97, *P* = 0.04), lower risk of intestinal obstruction (OR = 0.07, 95%CI = 0.01 to 0.43, *P* = 0.004), and higher risk of reflux esophagitis (OR = 2.47, 95%CI = 1.07 to 5.72, *P* = 0.03).

**Conclusion:**

The results of our study indicated that EG has significant advantages during the perioperative period and in short-term outcomes compared to JI.

## 1. Introduction

Proximal gastric cancer is characterized by large tumor size, high incidence of lymph node metastasis, strong invasive ability, and poor prognosis. The incidence of proximal gastric cancer has increased significantly in China in recent years [1]. Radical surgery is still the most effective cure, and the Japanese Gastric Carcinoma Association (JGCA) guidelines (14th edition) suggest that patients should accept D0, D1, and D1+ lymphadenectomy radical surgery, but the choice of reconstruction method is still a journal of concern issue. JGCA treatment guidelines indicate that proximal gastrectomy (PG) should only be performed for early gastric cancer, and at least half of the stomach should be preserved to maintain physiological function of the remnant stomach by open or laparoscopic surgery [2]. That could maintain the gastric reservoir with preservation of physiological function [3, 4] and improve postoperative quality of life [5]. There are various reconstruction methods after PG, such as esophagogastrostomy (EG), jejunal interposition (JI), jejunal pouch interposition (JPI), gastric tube reconstruction, and double tract (DT). EG has been widely used compared with the other reconstruction methods and is a simple and easy reconstruction method because it only has one anastomotic site [6].

JI reconstruction was first reported in 1946 and is associated with lower risk of reflux esophagitis [7]. Many authors stated that JI has significant short-term advantages. Katai et al. recommended that JI is an optimal treatment method with favorable long-term postoperative outcome [8]. Quite a few studies have reported that JI can reduce reflux esophagitis significantly and has diet tolerance with few complications [9].

EG and JI are used more frequently than other reconstruction methods. However, the standard method of reconstruction after PG is still controversial. Therefore, the purpose of this study was to compare the clinical efficacy of two reconstruction methods and to identify the advantages of EG and JI.

## 2. Materials and Methods

### 2.1. Study Selection

Search of Medline, Embase, J-STAGE, Cochrane Library, and PubMed databases identified retrospective series on EG and JI. We used the terms “gastrectomy,” “gastric cancer,” “esophagogastrostomy,” and “jejunal interposition” using [Mesh] or [free words]. The search was limited to January 1990 to January 2019.

### 2.2. Data Extraction

Two researchers (Nan D and Pei W) extracted the data independently. Final check was confirmed by the corresponding author. The data included the following parameters: operating time [10–14], blood loss [10–14], hospital stays [11–14], anastomotic leakage [11, 12, 14–17], anastomotic stenosis [11–17], intestinal obstruction [11, 12, 17], and reflux esophagitis [10–12, 14–17].

### 2.3. Inclusion Criteria

The following are the inclusion criteria: (1) diagnosis of the tumor as primary gastric cancer; (2) studies including clinical course such as operation-related data and complications; (3) studies including EG and JI; (4) availability of published data; (5) TNM stage lower than T3; and (6) adult population.

### 2.4. Exclusion Criteria

The following are the exclusion criteria: (1) gastric cancer was not the primary lesion; (2) case reports, letters, or meta-analyses; and (3) patients had severe underlying disease that may have affected treatment outcome.

### 2.5. Quality Assessment

Our meta-analysis included only retrospective cohort studies. Therefore, the Newcastle-Ottawa Scale (NOS) was used to analyze the quality of each study [18]. A cumulative score of NOS is according to three domains: the selection of study groups, comparability of cases, and ascertaining of the outcome. The scale of NOS is based on a 9-score model. Studies were considered having a high risk of bias (low quality) with scoring of less than three, medium risk of bias (moderate quality) if the score was four to six, and low risk of bias (high quality) if the score was seven to nine. Two researchers (Nan D and Pei W) assessed the trials independently. When opinions differed, the issue was resolved by the corresponding author.

### 2.6. Statistical Analysis

The data were analyzed using Review Manager Version 5.3 and Stata 11.0. Weight mean differences (WMDs), odds ratios (ORs), and 95% confidence intervals (CIs) were used to analyze the clinical outcomes and complications. Heterogeneity was measured with *I*^2^ index and *P* value [19]. Heterogeneity was regarded as significant with *I*^2^ > 50% or *P* value < 0.1. Due to inherent biases in retrospective study designs, the analyses were combined with the random-effects model. Potential publication bias was assessed with the Egger regression test. Sensitivity analysis was used to further assess the potential effect of heterogeneity by excluding one study at a time.

In this study, we followed the preferred reporting items, as stated in the systematic reviews and meta-analyses (PRISMA) [20].

## 3. Results

**Figure 1:**
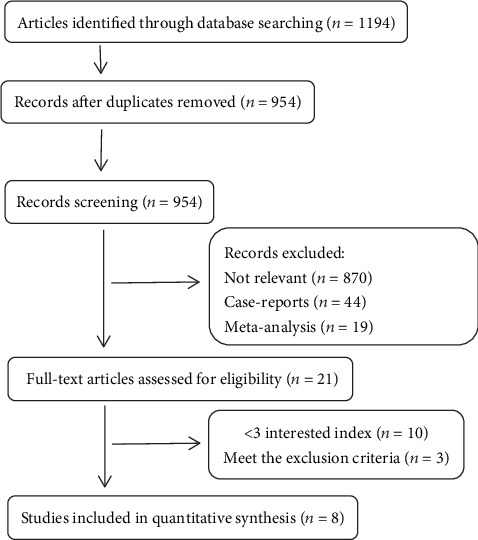
Flow diagram of the study selection process for meta-analysis.

A total of 3,194 studies were reviewed in our search (see Figure 1), and 2,114 articles were excluded because they were not relevant. Finally, we included eight relevant articles [10–17] with a total of 496 patients. Depending on NOS criteria, three studies were retrospective with medium risk of bias and five studies were considered high quality with low risk of bias (see Table 1).

## 4. Operation-Related Data

### 4.1. Operating Time

**Figure 2:**
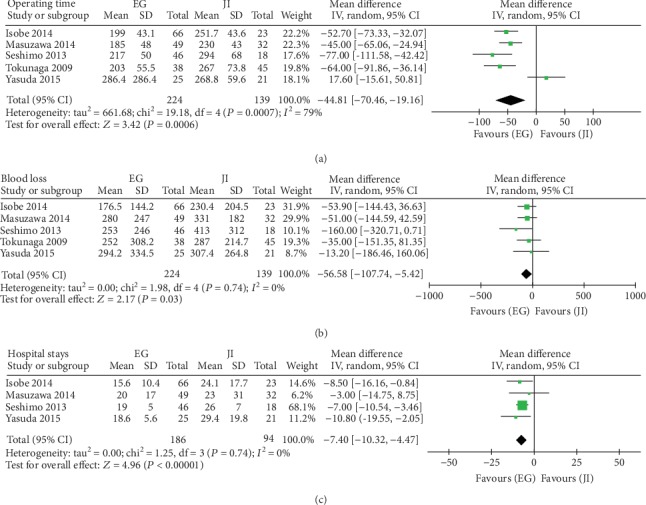
Meta-analysis of operative data on EG versus JI: (a) operative time (min), (b) blood loss (mL), and (c) postoperative hospital stays (days).

Five articles had available data on operating time; four of which demonstrated that EG had a shorter operating time than JI had (WMD = ‐44.81, 95%CI = ‐70.46 to‐19.16, *P* < 0.001). The heterogeneity between the groups was high in the random-effects model (*I*^2^ = 79%, *P* < 0.001) (see Figure 2(a)), which disappeared (*I*^2=^0, *P* = 0.40) when Yasuda 2015 trial was excluded; the WMD ranged from -44.81 (95% CI -70.46 to -19.16) to -54.96 (95% CI -66.95 to -42.98). The Egger test showed that there was no publication bias (*P* = 0.561).

### 4.2. Blood Loss

Five articles were used to compare blood loss between the groups. The JI and EG groups had a significant decrease in blood loss in the random-effects model (WMD = −56.58, 95%CI = ‐107.74 to‐5.42, *P* = 0.03). There was no heterogeneity (*I*^2^ = 0%, *P* = 0.74) (see Figure 2(b)). Sensitivity analyses showed no changing of heterogeneity by omitting one study at a time. The Egger test showed that there was no obvious potential publication bias (*P* = 0.655).

### 4.3. Hospital Stays

Five studies reported hospital stay. There was no significant heterogeneity between the groups (*I*^2^ = 0%, *P* = 0.74). In the EG group, hospital stay was 7.4 days shorter than in the JI group in the random-effects model (WMD = ‐7.40, 95%CI = ‐10.32 to‐4.47, *P* < 0.001) (see Figure 2(c)). Sensitivity analysis manifested no significant heterogeneity change. The Egger test showed no evidence of publication bias (*P* = 0.157).

## 5. Complications

### 5.1. Anastomotic Leakage

**Figure 3:**
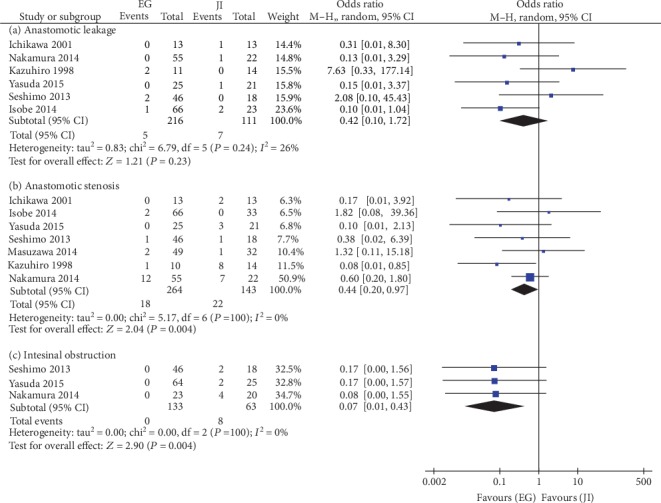
Meta-analysis of postoperative complications associated with EG versus JI: (a) anastomotic leakage, (b) anastomotic stenosis, and (c) intestinal obstruction.

Six articles reported anastomotic leakage, but there was no significant difference between the two groups in the random-effects model (OR = 0.42, 95%CI = 0.10 to 1.72, *P* = 0.23) with low heterogeneity (*I*^2^ = 26%, *P* = 0.24) (see Figure 3(a)). Sensitivity analysis showed no heterogeneity changing. There was no significant publication bias (*P* = 0.383).

### 5.2. Anastomotic Stenosis

Seven articles reported anastomotic stenosis. The incidence of anastomotic stenosis in the JI group was higher than that in the EG group in the random-effects model (OR = 0.44, 95%CI = 0.20 to 0.97, *P* = 0.04). There was no heterogeneity (*I*^2^ = 0%, *P* = 0.52) (see Figure 3(b)) and no publication bias between the two groups (*P* = 0.460). Sensitivity analysis for this parameter showed no significant change when a single study was removed.

### 5.3. Intestinal Obstruction

Three articles included data on intestinal obstruction. The JI group had a significant 91% increase in the risk of intestinal obstruction in the random-effects model (OR = 0.07, 95%CI = 0.01 to 0.43, *P* = 0.004), and no heterogeneity was present (*I*^2^ = 0%, *P* = 1.00) (see Figure 3(c)). Sensitivity analysis demonstrated no heterogeneity changing. The studies to assess the publication bias were not enough.

### 5.4. Reflux Esophagitis

**Figure 4:**
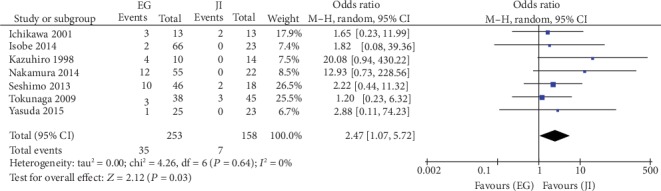
Meta-analysis of postoperative complications associated with EG versus JI: reflux esophagitis.

Six studies reported the outcomes of EG and JI after PG. In the random-effects model, the EG group had a higher risk of reflux esophagitis than the JI group had (OR = 2.47, 95%CI = 1.07 to 5.72, *P* = 0.03). Among the trials, there was no heterogeneity (*I*^2^ = 0, *P* = 0.64) (see Figure 4) or publication bias (*P* = 0.093). Sensitivity analyses showed that the overall effects remained similar by excluding the trials by turns.

## 6. Discussion

PG has been used worldwide, and postoperative reconstruction methods are controversial. The JGCA recommends that early gastric cancer can be treated by PG. Nevertheless, indications for surgery of proximal gastric cancer are unclear in the National Comprehensive Cancer Network guidelines [21]. Tsuji et al. claimed that EG is used for resection of less than one-third of the stomach [22]. In contrast, other authors have stated that JI is a superior reconstruction method compared with EG [23]. It remains unclear as to which type of reconstruction is most effective after PG.

We performed a meta-analysis to compare the postoperative complications between EG and JI. Compared to JI, in the EG group, operating time and hospital stay were shorter and there was less blood loss. Furthermore, EG also had the advantage of technical simplicity, which reduced surgical difficulty and increased patient safety. The EG group had a lower risk of anastomotic stenosis and intestinal obstruction compared with the JI group, but the EG group had a higher risk of postoperative reflux esophagitis. We demonstrated that EG had significant short-term efficacy.

EG is related to the high postoperative risk of reflux esophagitis, and it has been shown that gastroesophagitis after PG occurs in 10–30% of patients [24]. Nowadays, modified EG has been regarded as a simple, less-invasive procedure because it has benefits to complications and outstanding antireflux function. However, the optimal modification of EG still needs more research. Proton pump inhibitors can control reflux esophagitis, but the effect is not satisfactory. In our study, there were three studies of modified EG, which combined EG with pyloroplasty [15], the gastric tube with the angle of His [12], and fundoplication [17]. These studies all supported the superiority of modified EG for antireflux activity. Adachi et al. found that the symptoms of reflux esophagitis after gastric tube reconstruction occur only rarely [25]. Someya et al. confirmed that duodenal switch after PG could be the preferred surgical treatment for reflux gastroesophagitis because this procedure is less invasive and alleviates the patient's symptoms [26]. Some other types of reconstruction also play significant roles in the antireflux function. Fundoplication and pyloroplasty have proven to be effective procedures for preventing reflux esophagitis after EG and increase the quality of life. Shada et al. suggested that pyloroplasty can be regarded as a safe and effective treatment method with low morbidity [27]. In particular, Nissen fundoplication can preserve antireflux function better than Toupet fundoplication can [28].

In recent years, laparoscopic distal and total gastrectomy has become widely accepted and has crucial advantages in comparison with open procedures in the treatment of early gastric cancer, such as less intraoperative blood loss, faster resumption of gastrointestinal function, and reduced postoperative morbidity [29, 30]. Few studies have focused on laparoscopic PG due to its technical difficulty. In our study, only one study used a laparoscopy-assisted technique. This simple procedure combines a gastric tube with the angle of His, which can preserve the quality of life. Laparoscopic gastrectomy carries a lower risk of inflammatory reactions in Asian gastric cancer patients [31]. Although laparoscopy-assisted PG has advantages in short-term outcomes for early gastric cancer, the results should be confirmed by more clinical trials.

D2 total gastrectomy has been considered the standard procedure for the treatment of gastric cancer worldwide. In recent decades, PG has frequently been performed in China and Japan to preserve the physiological function for maintaining the gastric reservoir for early proximal gastric cancer. Some authors advocated functional advantages of PG with JI over total gastrectomy with Roux-en-Y EG [32]. By contrast, in western countries, no consensus has been reached on the reconstruction of proximal gastric cancer. Rosa et al. claimed that PG might increase the mortality rate and risks of complications [33]. PG has been performed in patients with advanced gastric cancer, although some still prefer total gastrectomy. In previous retrospective studies, many clinical parameters, such as cancer stage, body mass index, surgical outcome, and frequent postoperative complications, were not included, and these issues need to be considered.

At present, there are several methods of reconstruction of the alimentary tract after PG. In addition to EG and JI, there are many other reconstruction methods like JPI and DT. A multitude of studies has reported that JPI is comparatively easy and can improve the postoperative quality of life compared to single JI [23, 34, 35]. Nakamura et al. clarified that, in comparison to JI and JPI, EG had benefits of lower invasiveness. Additionally, a host of studies have suggested that DT reconstruction has a lower incidence of postoperative complications than EG has especially reflux esophagitis [36, 37]. However, its superiority needs more long-term clinical data to confirm.

The limitations of the present study were as follows. First, the heterogeneity of operating time was significant (*I*^2^ = 79%). That might have been the result of different surgical techniques of the surgeons and different surgical equipment of the hospital. We conducted a sensitivity analysis to assess the potential effect of heterogeneity of operating time and found the Yasuda 2015 trial could be the major originator after excluded (the *I*^2^ ranged from 79% to 0). We further compared the Yasuda 2015 trial with extra included trials. We found that the surgical technique of the EG group was modified by creating the new cardiac notch (angle of His). This complex procedure might need more operating time to finalize, and it could be one of the main reasons for high heterogeneity. Second, the trials included in our study all had short-term outcomes, and long-term overall survival is still controversial. Third, on account of not enough studies, we could not assess the evidence of publication bias on the trials of intestinal obstruction. Furthermore, we used the random-effects model to replace the fixed-effects model when the heterogeneity was significant. Moreover, our meta-analysis only included EG and JI, and there are many other types of reconstruction; however, there is no comprehensive study to clarify the optimal reconstructive procedure after PG.

## 7. Conclusions

In conclusion, our study indicated that EG had significant advantages during the perioperative period and for short-term outcomes compared to JI. Moreover, EG combined with fundoplication reduced the risk of complications and improved the quality of life. However, the overall survival and long-term prognosis after PG should be confirmed by large multicenter clinical trials with longer follow-up.

## Figures and Tables

**Table 1:** Clinical characteristic of the included studies in meta-analysis.

Authors	Years	Design	Quality score	Group	No. of patients	Age (mean)	Gender (M/F)	Population
Seike et al. [15]	1998	Retrospective	5	EG	11	69.3	10/1	EGC
					JI	14	54.8	8/6	

Ichikawa et al. [16]	2001	Retrospective	5	EG	13	N/A	N/A	EGC
					JI	13	N/A	N/A	

Tokunaga et al. [10]	2009	Retrospective	6	EG	36	63.6	30/6	EGC/AGC
					JI	40	60.9	31/9	

Seshimo et al. [11]	2013	Retrospective	7	EG	46	64.8	36/10	EGC/AGC
					JI	18	68.0	13/5	

Yasuda et al. [12]	2015	Retrospective	7	EG	25	71.6	18/7	EGC
					JI	21	61.0	13/8	

Masuzawa et al. [13]	2014	Retrospective	9	EG	49	64.0	36/13	EGC
					JI	32	65.0	25/7	

Isobe et al. [14]	2014	Retrospective	8	EG	66	71.6	52/14	EGC/AGC
					JI	23	59.4	18/5	

Nakamura et al. [17]	2014	Retrospective	8	EG	64	73	49/15	EGC
					JI	25	70	21/4	

## Data Availability

The data supporting this meta-analysis are from previously reported studies and datasets, which have been cited. The processed data are available from the corresponding author upon request.

## Supplementary Materials

Supplementary Materials Table S1: risk of bias assessment in those 8 observational studies. Figure S1: Egger's publication bias plot of operating time. Figure S2: Egger's publication bias plot of blood loss. Figure S3: Egger's publication bias plot of hospital stays. Figure S4: Egger's publication bias plot of anastomotic leakage. Figure S5: Egger's publication bias plot of anastomotic stenosis. Figure S6: Egger's publication bias plot of reflux esophagitis. Figure S7: age moderator metaregression analyses for reflux esophagitis in EG vs. JI. Figure S8: gender moderator metaregression analyses for reflux esophagitis in EG vs. JI.
