# Supplementary material for: Reconstruction Methods and Complications of Esophagogastrostomy and Jejunal Interposition in Proximal Gastrectomy for Gastric Cancer: A Meta-Analysis
Source: Gastroenterol Res Pract. 2020 Jan 16;2020:8179254. doi: 10.1155/2020/8179254 (PMC7201443; doi:10.1155/2020/8179254)
Supplement: Supplementary Materials — Table S1: risk of bias assessment in those 8 observational studies. Figure S1: Egger's publication bias plot of operating time. Figure S2: Egger's publication bias plot of blood loss. Figure S3: Egger's publication bias plot of hospital stays. Figure S4: Egger's publication bias plot of anastomotic leakage. Figure S5: Egger's publication bias plot of anastomotic stenosis. Figure S6: Egger's publication bias plot of reflux esophagitis. Figure S7: age moderator metaregression analyses for reflux esophagitis in EG vs. JI. Figure S8: gender moderator metaregression analyses for reflux esophagitis in EG vs. JI. [file 8179254.f1.docx]

Table S1 Risk of bias assessment in those 8 observational studies

| **Study** **[Ref.]** | **Selection** | **Comparability** | **Outcome ascertainment** | **Bias risk** **(Total scoring)** |
| --- | --- | --- | --- | --- |
| Seike [13] | 2 | 0 | 3 | 5 |
| Ichikawa [14] | 2 | 2 | 1 | 5 |
| Tokunaga [15] | 3 | 1 | 2 | 6 |
| Seshimo [16] | 3 | 2 | 2 | 7 |
| Yasuda [17] | 2 | 3 | 2 | 7 |
| Masuzawa [18] | 4 | 3 | 2 | 9 |
| Isobe [19] | 4 | 2 | 2 | 8 |
| Nakamura [20] | 3 | 2 | 3 | 8 |


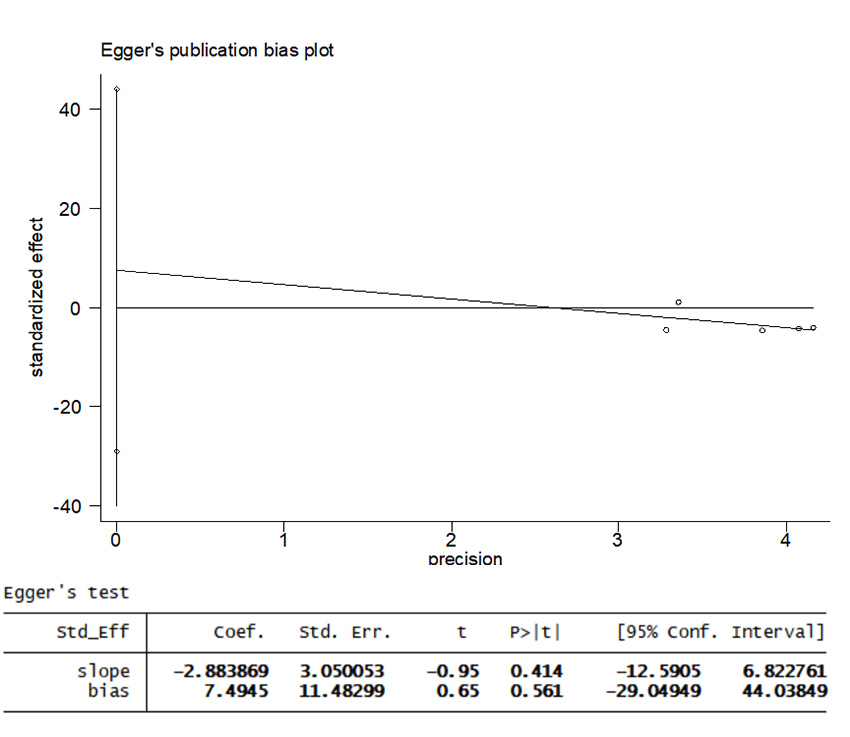


**Figure S1: Egger’s publication bias plot of operating time**


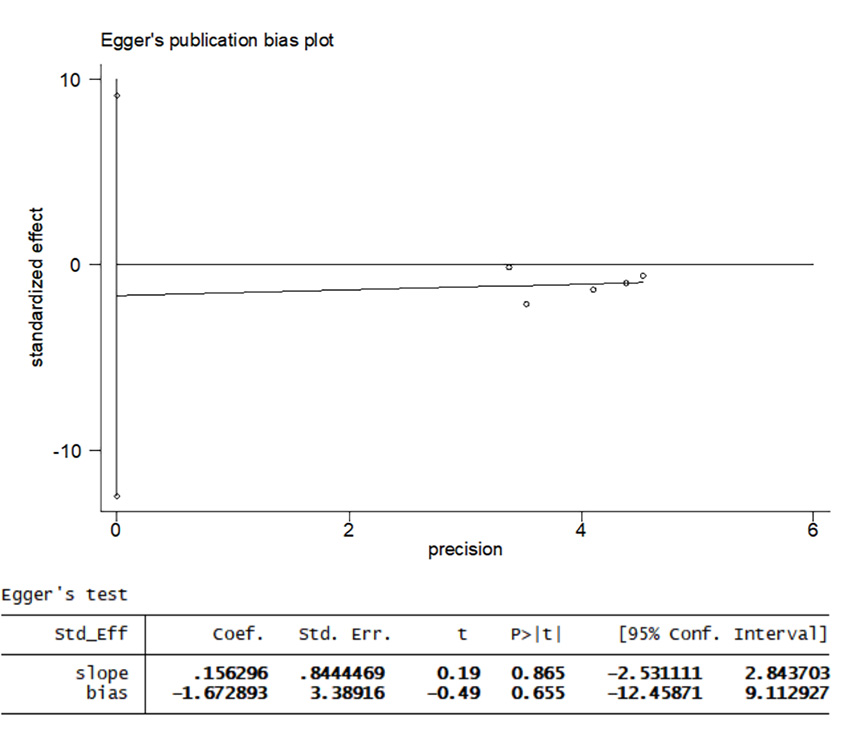


**Figure S2: Egger’s publication bias plot of blood loss**


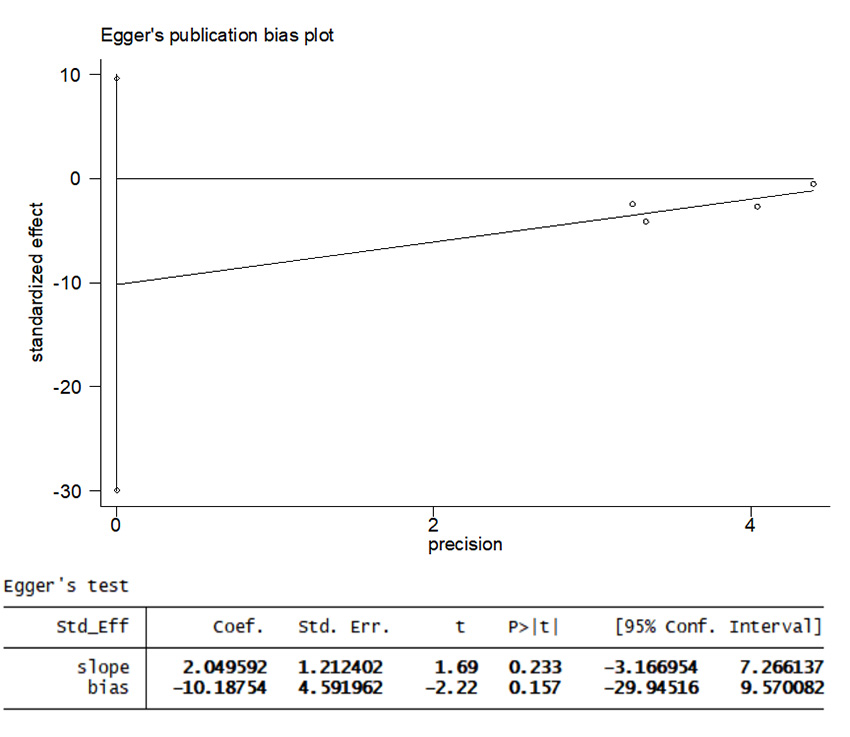


**Figure S3: Egger’s publication bias plot of hospital stays**


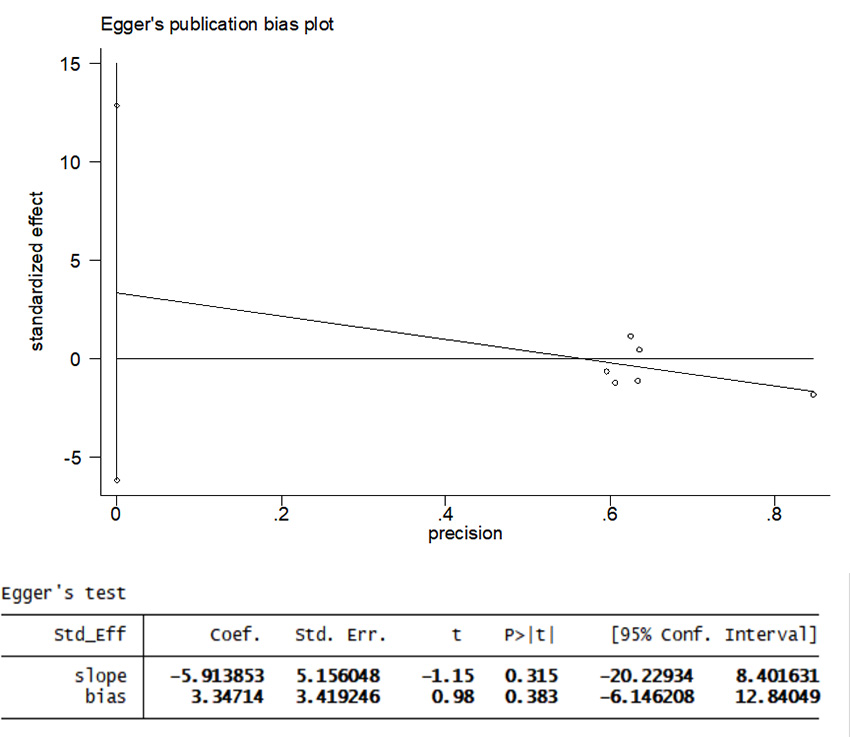


**Figure S4: Egger’s publication bias plot of anastomotic leakage**


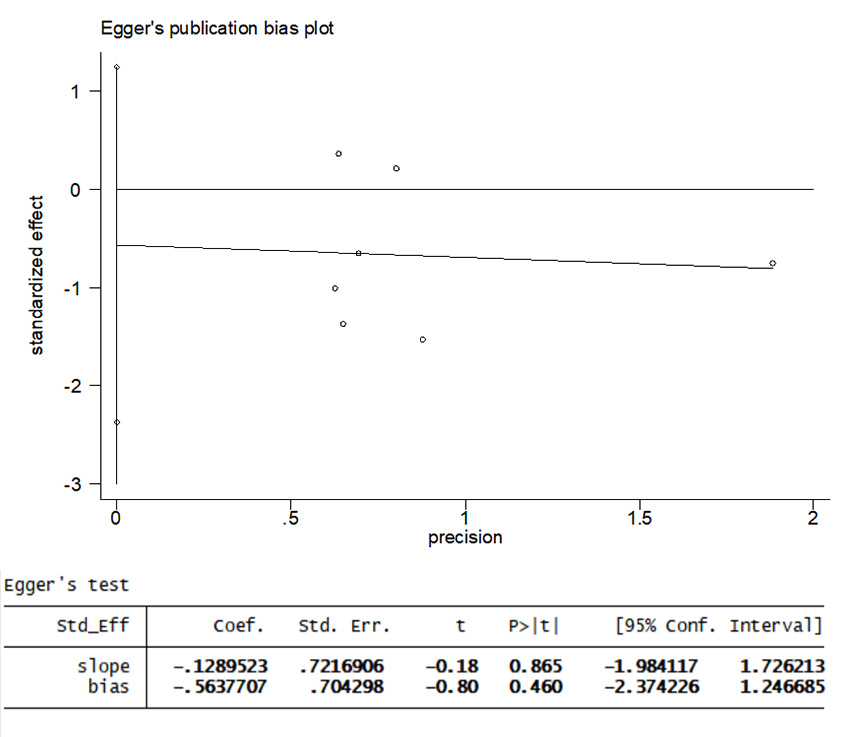


**Figure S5: Egger’s publication bias plot of anastomotic stenosis**


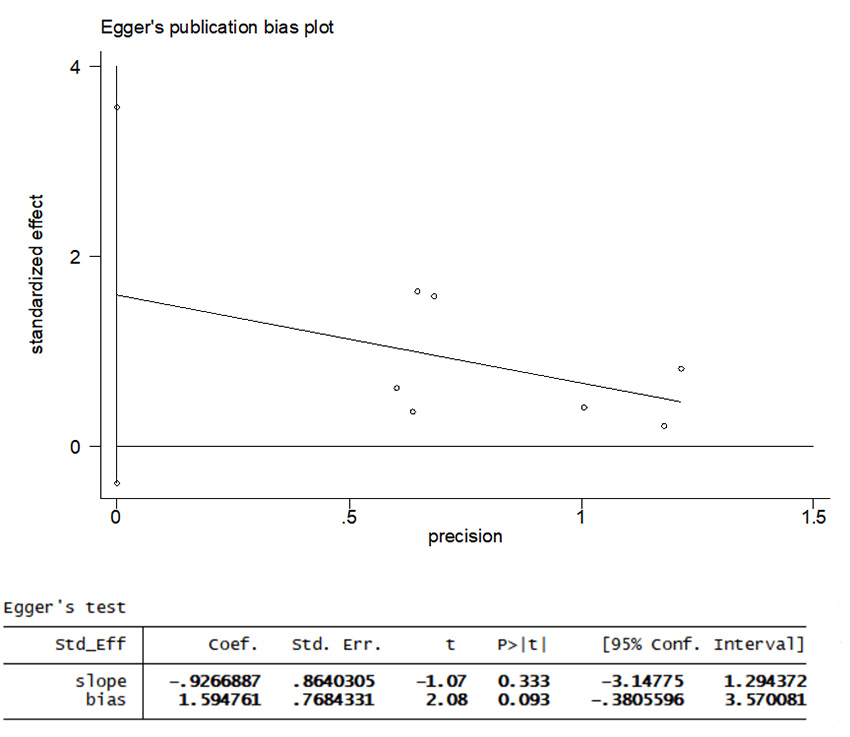


**Figure S6: Egger’s publication bias plot of reflux esophagitis**


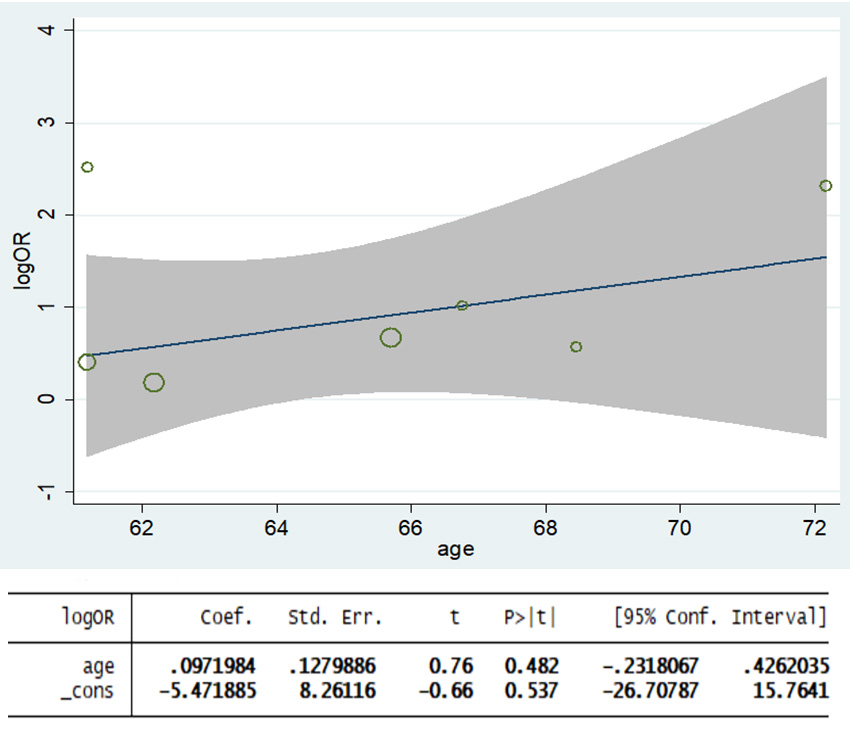


**Figure S7: Age moderator meta-regression analyses for reflux esophagitis in EG vs. JI**


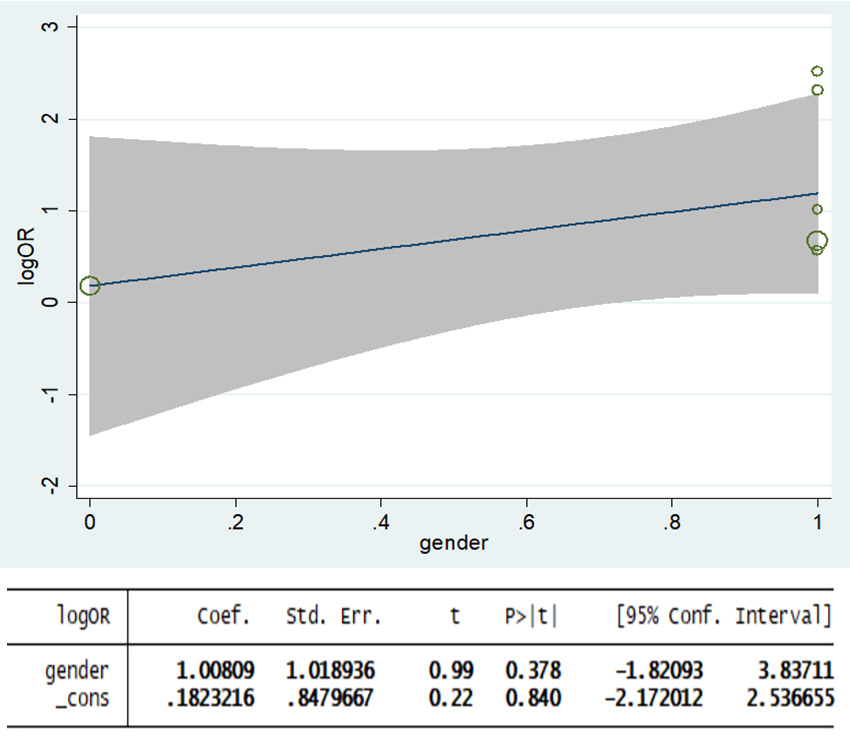


**Figure S8: Gender moderator meta-regression analyses for reflux esophagitis in EG vs. JI**
